# Restoring a sense of safety among survivors of sexual harassment in higher education: policy recommendations from the UNI4EQUITY multinational study

**DOI:** 10.3389/fsoc.2026.1782978

**Published:** 2026-03-02

**Authors:** Sylwia Jaskulska, Barbara Jankowiak, Vanesa Perez-Martrnez, Stefano Porru, Angela Carta, Marlies Wallner, Viktoria Stifter, Joana Topa, Iwetta Andruszkiewicz, Katarzyna Waszyńska, Aitana Munoz-Haba, Jose Miguel Carrasco, Carmen Vives-Cases

**Affiliations:** 1 Faculty of Educational Studies, Adam Mickiewicz University in Poznan, Poznan, Poland; 2 Department of Community Nursing, Preventive Medicine and Public Health and History of Science, University of Alicante, Alicante, Spain; 3 Department of Diagnostics and Public Health, University of Verona, Verona, Italy; 4 Department of Health and Social Sciences, University of Applied Sciences Burgenland, Eisenstadt/Pinkafeld, Austria; 5 Department of Social and Behavioural Sciences, Universidade da Maia, Maia, Portugal; 6 Faculty of Political Science and Journalism, Adam Mickiewicz University in Poznan, Poznan, Poland; 7 APLICA Cooperative, Madrid, Spain; 8 CIBER of Epidemiology and Public Health (CIBERESP), Madrid, Spain

**Keywords:** higher education, institutional trust, restoring a sense of safety, sexual harassment, university climate

## Abstract

Sexual harassment (SH) is a global public health concern that remains widespread in higher education, affecting individuals, groups, and institutions. This study, based on the UNI4EQUITY multinational survey involving 7,563 participants from six European universities, offers policy recommendations aimed at enhancing institutional safety for survivors. The results indicate a 37% prevalence of SH, occurring both offline and online. Among individuals with SH experience, women, LGB persons, and those aged 25–39 report lower perceptions of university safety. Trust in institutional support networks is positively associated with perceived safety, whereas reporting SH is linked to decreased feelings of safety. These findings highlight the need for a multi-level, systemic response, including awareness-raising, transparent governance, accessible support services, clear reporting mechanisms, and effective survivor protection to promote safety, trust, and institutional accountability.

## Introduction

Sexual harassment (SH), recognized as a global public health concern, remains a widespread problem in higher education despite ongoing efforts to address it, affecting individuals, groups, and institutions alike (Bondestam and Lundqvist, 2020). The European Institute for Gender Equality (EIGE) defines SH as any unwanted verbal, non-verbal, or physical sexual behavior that violates a person’s dignity or creates a hostile, degrading, or offensive environment, including cyber-harassment (Walker et al., 2019). SH in higher education (HE) has serious consequences for both individuals and institutions. Exposure to SH leads to a range of physical, psychological, and professional consequences, such as stress, discomfort, feelings of powerlessness and humiliation, depression, symptoms of PTSD, sexually transmitted infections, limited career opportunities, and reduced job motivation. Institutions, in turn, face substantial costs related to staff turnover, recruitment, and complaint investigations (Bondestam and Lundqvist, 2020). Moreover, SH functions as an independent occupational stressor that cumulatively undermines well-being and functioning (Sojo et al., 2015).

Organizational climate refers to the shared perceptions within a community and plays a key role in shaping behavior in organizations (Walker et al., 2019). Although definitions vary, researchers agree that identifying the factors contributing to a safe climate can help universities reduce experiences that undermine students’ and staff members’ well-being and success (Minnotte and Pedersen, 2023; Moylan et al., 2021). A safe organizational climate is crucial not only for preventing SH but also for supporting individuals who have experienced it. When universities foster a culture that is intolerant of SH and actively address the structural and relational norms that sustain it, they help strengthen survivors’ coping mechanisms, resilience, and sense of security (Ford and Ivancic, 2020). Conversely, the mishandling of complaints undermines trust in reporting systems and discourages victims from seeking help (Vijayasiri, 2008). A safe and responsive climate, where disclosures are met with empathy, credibility, and tangible support, can significantly influence recovery and professional stability (Ford and Ivancic, 2020; Vijayasiri, 2008). While informal support from colleagues within an organization is often perceived as helpful, formal interventions are frequently viewed as overly cautious and skeptical (Lorenz and O’Callaghan, 2022). Therefore, creating a trustworthy, transparent, and survivor-centered climate is essential for survivors’ well-being and for cultivating a healthier, more equitable academic environment. Specifically, prior findings suggest that organizational culture shapes survivors’ coping, resilience, and vulnerability (Ford and Ivancic, 2020).

## Methods

Data on university climate was obtained from a multinational study on SH conducted among students, academic and teaching staff, and administrative personnel as part of the UNI4EQUITY project (CERV-2022-DAPHNE GA: 10109412) (Vives-Cases et al., 2025). The aim of the study was to assess the capacity of European universities to respond to sexual violence and to inform preventive measures in the academic environment. Data was collected between November 2023 and March 2024. The project involved a consortium of six universities—University of Alicante (UA), University of Antwerp (UAntwerp), Adam Mickiewicz University (AMU), University of Maia (UMAIA), University of Applied Sciences Burgenland (UASB), and University of Verona (UNIVR)—as well as two research entities with expertise in gender, sexual health, and violence prevention: Centro Studi e Iniziative Europeo – Ente del Terzo Settore (CESIE ETS) and Cooperative APLICA (Vives-Cases et al., 2025). Ethical approval was granted by the respective university research ethics committees: University of Alicante (Vice-Rectorate for Research, Ref. no. UA-2023-03-27); University of Verona (Comitato di Approvazione per la Ricerca sulla Persona, Ref. no. UNIVR-24/2023); University of Antwerp (Ethics Committee for the Social Sciences and Humanities, Ref. no. EX_SHW_2023_38_1); Adam Mickiewicz University (Ethics Committee for Research Involving Human Participants, Ref. no. UAM_19/2022/2023); University of Applied Sciences Burgenland (Ethics Committee, Ref. no. UASB_28/08/2023); University of Maia (Conselho de Ética e Deontologia, Ref. no. UMAIA_151/2023) (Vives-Cases et al., 2025).

Data were collected from 7,563 participants, predominantly women (sex assigned at birth: men 29.6%, women 70.4%). The largest age group was 20–24 (36.9%) years, followed by 25–39 years (23.6%) and 40 years or older (26%), and 25–39 years (23.6%). The smallest group was participants aged 19 or younger (13.6%). Regarding university positions, the sample consisted of students (64.3%), research staff, residents, professors, lecturers (21.4%), and administrative and support services staff (14.8%). Most respondents identified as heterosexual (83%), while 17% identified as LGB+. Participants reported being in both steady (59.1%) and non-steady (49.9%) relationships (respondents could select more than one option). The majority (96.1%) did not identify as belonging to an ethnic minority, while 3.9% did. Perceived socioeconomic status was most often rated as medium to high (84.7%), while 15.3% rated it as low.

Data were analyzed using Ordinal Logistic Regression (OLR), adjusting for sociodemographic variables (Table 1) and perceived institutional response to SH (perception of institutional capacity to deal with SH, general help seeking, knowledge of the process or resources to report SH, trust and official reporting SH) (Table 2). The analyses included only individuals who had experienced SH, either in person or online.

**Table 1:** Model 1 sociodemographic characteristics – ordinal logistic regression for perceived SH climate at the university (*N* = 1,289).

Sociodemographic variables	Model 1 sociodemographic characteristics					
	IRR	Std. err.	*z*	*p* > *z*	CI 95%	
Sex at birth (ref: men)						
Women	0.509	0.102	−3.360	0.001	0.343	0.755
Age (ref: 19 or less)						
20 to 24	0.968	0.261	−0.120	0.904	0.571	1.642
25 to 39	0.523	0.170	−2.000	0.046	0.276	0.989
40 and more	0.504	0.203	−1.700	0.090	0.229	1.112
Sexual orientation (ref: heterosexual)						
LGB	0.626	0.101	−2.890	0.004	0.456	0.860
Relationship status (ref: not in a steady relationship)						
Steady relationship	1.103	0.162	0.660	0.506	0.827	1.471
Ethnic minority (ref: no)						
Yes	0.614	0.169	−1.770	0.077	0.358	1.055
Socioeconomic level (ref: low)						
Medium-high	1.525	0.274	2.350	0.019	1.072	2.168
Position at university (ref: undergraduate student)						
Graduate student	0.875	0.254	−0.460	0.646	0.496	1.546
Research staff or resident	0.789	0.219	−0.850	0.393	0.458	1.359
Professor or lecturer	1.284	0.456	0.700	0.482	0.640	2.575
Staff working in administration or services	1.227	0.413	0.610	0.544	0.634	2.372

**Table 2:** Model 2 perceived response of the university to SH – ordinal logistic regression for perceived SH climate at the university (*N* = 427).

Sociodemographic variables	Model 2 sociodemographic characteristics + perceived response of the university to SH					
	IRR	Std. err.	*z*	*p* > *z*	CI 95%	
Sex at birth (ref: men)						
Women	0.514	0.200	−1.710	0.086	0.240	1.100
Age (ref: 19 or less)						
20 to 24	1.176	0.678	0.280	0.779	0.380	3.642
25 to 39	0.810	0.531	−0.320	0.749	0.224	2.930
40 and more	0.960	0.780	−0.050	0.960	0.195	4.724
Sexual orientation (ref: heterosexual)						
LGB	0.727	0.224	−1.040	0.301	0.398	1.329
Relationship status (ref: not in a steady relationship)						
Steady relationship	1.621	0.427	1.830	0.067	0.967	2.716
Ethnic minority (ref: no)						
Yes	1.017	0.492	0.030	0.973	0.394	2.625
Socio-economic level (ref: low)						
Medium-high	1.818	0.568	1.910	0.056	0.985	3.355
Position at university (ref: undergraduate student)						
Graduate student	1.094	0.564	0.170	0.861	0.398	3.008
Research staff or resident	2.106	1.037	1.510	0.130	0.802	5.528
Professor or lecturer	1.993	1.327	1.040	0.300	0.540	7.353
Staff working in administration or services	1.601	0.975	0.770	0.440	0.485	5.280
Assessment of institutional capacity to deal with SH	1.030	0.019	1.630	0.103	0.994	1.068
General help seeking after SH and sexual violence (SV) (ref: no)						
Yes	0.845	0.265	−0.540	0.592	0.457	1.562
For some experiences, I did tell them to someone, and for other experiences, I did not.	0.877	0.290	−0.400	0.692	0.459	1.677
Knowledge of the process or resources to report SH (ref: no knowledge)						
Yes, at least one	1.195	0.346	0.620	0.539	0.678	2.107
Trust in the closest help in case of SH victimization	1.110	0.044	2.650	0.008	1.028	1.200
Trust in the university or people in case of a SH situation	1.450	0.112	4.820	0.000	1.247	1.686
Official reporting SH and SV at university resources, or elsewhere (ref: no, they did not report)						
Yes, they reported (*n* = 90)	0.240	0.105	−3.260	0.001	0.102	0.565

## Results

The results indicate that the overall prevalence of SH – whether online or in person – was 37% among respondents, with 20% reporting such experiences within the past 12 months. In-person SH was more commonly reported than online SH: 31% of respondents had experienced in-person SH since starting at the university and 16% within the last year, compared to 17 and 8%, respectively, for online SH.

Among individuals who had experienced SH, being female (compared to men) (IRR [95% CI]: 0.509 [0.343–0.755]), having between 25–39 years (compared to <19 years) (IRR [95% CI]: 0.523 [0.276–0.989]), and having LGB orientation (compared to heterosexual orientation) (IRR [95% CI]: 0.626 [0.456–0.860]) was associated with a lower likelihood of perceiving the university climate as safe in terms of the perceived risk of SH. On the other hand, having a medium-high socieconomical level was associated with a higher probability of perceiving the university climate as safe, compared to people with low socioeconomical level (IRR [95% CI]: 1.525 [1.072–2.168]) (Table 1).

People who have a medium-high socieconomical level (compared with those who have lower socioeconomical level) were associated with a higher probability of perceiving the university climate as safe, compared to people with low socioeconomical level (IRR [95% CI]: 1.818 [0.985–3.355]). Trust in the closest (e.g., classmates or colleagues) to provide help in case of SH victimization, for instance, by offering information on how to deal with the incident and/or providing emotional support, was associated with a higher likelihood of perceiving the university climate as safe (IRR [95% CI]: 1.110 [1.028–1.200]). Similarly, trust in the university and its community in situations involving SH, such as believing that university officials will adequately protect victims and that peers will report those who engage in harassment, was also positively associated with perceived safety (IRR [95% CI]: 1.450 [1.247–1.686]). Contrary, reporting experiences of SH and sexual violence was associated with a lower likelihood of feeling safe at the university (IRR [95% CI]: 2.40 [0.102–0.565]).

These findings highlight the importance of institutional trust, perceived fairness, and leadership accountability as central components of safety in higher education settings. Building on these insights, the following policy recommendations outline practical strategies to strengthen institutional responses to SH.

## Discussion

The findings confirm that sexual harassment remains a widespread experience in higher education, with over one third of respondents reporting SH. This prevalence is consistent with previous systematic reviews showing that SH is a structural and persistent problem in academic institutions rather than isolated incidents (Bondestam and Lundqvist, 2020).

Our findings indicate that among those who have experienced SH, women, LGB individuals, and participants aged 25–39 perceive the atmosphere at the university as significantly less safe. This aligns with earlier research indicating that marginalized groups experience higher vulnerability and cumulative psychosocial consequences of harmful workplace experiences (Sojo et al., 2015).

The lower perception of safety among these individuals may reflect both greater exposure to sexual harassment and a sense of belonging to groups that are already disadvantaged at the University (European Commission: Directorate-General for Research and Innovation, 2021; Raja et al., 2024).

The key finding of this study is the strong link between trust in institutions and restoring a sense of security to people who have experienced SH. Trust in colleagues and in the university as an institution was positively linked to a safer climate, which supports previous evidence that organizational culture and psychological safety play a crucial role in shaping reporting behaviors and recovery processes (Vijayasiri, 2008; Walker et al., 2019). In line with Ford and Ivancic (2020), our findings suggest that a supportive climate can foster resilience, whereas institutional tolerance or ambiguity may lead to harassment fatigue and withdrawal.

Notably, official reporting of SH was associated with lower perceived safety. This paradox reflects earlier qualitative findings showing that formal procedures are often experienced as emotionally burdensome and insufficiently supportive (Lorenz and O’Callaghan, 2022). Together, these results indicate that reporting mechanisms alone are insufficient if they are not embedded in a transparent, empathetic and survivor-centered institutional culture.

## Policy recommendations

Preventing and addressing SH in European universities aligns with several equality frameworks, including the EU Gender Equality Strategy (2022–2025) and the 2019 International Labour Organization Convention. Higher education institutions must foster a workplace atmosphere that does not tolerate SH and take active measures to restore a sense of safety for those who have experienced SH or sexual violence (SV).

According to our research, supporting individuals affected by SH begins with building an institutional climate based on trust in the university, its leaders, and colleagues. Therefore, prevention should be approached at three levels: primary, secondary, and tertiary.

### Primary prevention

- Conduct a regular, evidence-based diagnosis of SH using mixed methods to understand its scope and patterns. Surveys, desk reviews, and stakeholder interviews should identify risk factors and vulnerable groups and assess levels of institutional trust and perceived fairness. Such data help identify *grey areas* and vulnerable groups requiring particular attention.
- Increase public awareness about rejecting all forms of SH. Social media campaigns and educational workshops play a crucial role in informing university communities and promoting safe and respectful environments. Information campaigns should highlight not only available resources but also the actions taken by university representatives to build a culture of support and trust.
- Integrate trust-building into leadership training, emphasizing transparent communication, fairness, and responsiveness to complaints.

### Secondary prevention

- Develop bystander intervention and peer-support programs that empower students and staff to recognize, prevent, and respond to SH. These initiatives strengthen a culture of safety, accountability, and trust.
- Ensure that information about reporting channels, support services, and digital tools is clearly communicated and regularly updated.
- Offer targeted support measures for groups at higher risk of SH, such as women and LGBTQ+ individuals. Gender-sensitive and empowerment-oriented actions can help reduce exposure to harassment.

### Tertiary prevention

- Provide training programs and workshops that equip community members with the skills to respond safely and effectively to both victims and perpetrators. These programs should promote inclusive and sensitive reactions to individuals of diverse genders, ages, and sexual orientations. Training on responding to SH should be particularly accessible to university leaders and student organization representatives.
- Guarantee specialized, free psychological support for survivors, delivered by professionals trained in trauma-informed, gender-sensitive, and intersectional approaches. These services should provide a safe and confidential space to receive emotional support, rebuild a sense of security, and access further assistance.
- Establish and communicate clear, survivor-centered reporting procedures with defined protection measures, especially in cases involving power hierarchies (e.g., supervisor–student).
- Ensure consistent perpetrator accountability through fair and transparent disciplinary processes and communicate outcomes to reinforce institutional trust.

## Conclusion

Restoring a sense of safety in universities requires building institutional trust and ensuring transparent, fair, and accountable responses to SH. Regular climate assessments, awareness campaigns, and leadership training focused on fairness and responsiveness should form the foundation of prevention. Accessible reporting channels, trauma-informed support, and targeted measures for vulnerable groups are essential to rebuilding confidence in institutional protection. Ultimately, protecting survivors and holding perpetrators accountable are key to creating a culture of trust, safety, and integrity in higher education.
